# Detection of Astrovirus in Historical Cases of European Sporadic Bovine Encephalitis, Switzerland 1958–1976

**DOI:** 10.3389/fvets.2016.00091

**Published:** 2016-10-11

**Authors:** Senija Selimovic-Hamza, Ilias G. Bouzalas, Marc Vandevelde, Anna Oevermann, Torsten Seuberlich

**Affiliations:** ^1^DCR-VPH, Division of Neurological Sciences, NeuroCenter, University of Bern, Bern, Switzerland; ^2^Graduate School for Cellular and Biomedical Sciences, University of Bern, Bern, Switzerland

**Keywords:** astrovirus, cattle, encephalitis, neurological disease, historical, archive

## Abstract

European sporadic bovine encephalitis is a frequent diagnosis in neurologically diseased cattle, but its etiology remained unresolved. Using *in situ* hybridization, we have detected a recently discovered neurotropic bovine astrovirus in historical tissues in a high proportion of brain samples of affected cattle. Our results suggest that astroviruses were already involved in the pathogenesis of the disease several decades ago, but have gone undetected.

## Introduction

In the 1930s, Frauchiger and Hofmann observed cases of non-suppurative poliencephalitis in adult cattle in Switzerland (1), but their first detailed description was only published in 1961 by the veterinary neuropathologist Rudolf Fankhauser, who named it sporadic bovine encephalitis (2). Since then, this disease has continuously been diagnosed in cattle with neurological disorders, but its etiology remained enigmatic (3–6). In 1998, Theil et al. reviewed 50 Swiss cases, excluding *Chlamydia* spp. as a potential etiology, which had been postulated for many years in analogy to sporadic bovine encephalomyelitis in the USA, and renamed the condition European sporadic bovine encephalitis (ESBE) (6).

Metagenomics-based approaches to virus discovery have led to new insights into so far unresolved neurological diseases in cattle. In 2013, Li and colleagues reported a divergent astrovirus associated with neurological disease in cattle in the USA, termed bovine astrovirus (BoAstV) NeuroS1 (7). At the same time, we found a closely related virus, BoAstV CH13, in brain tissues of cattle with viral encephalitis in Switzerland (8). In Switzerland, about one quarter of recent cases with the pathological diagnosis of non-suppurative bovine encephalitis were found positive for BoAstV CH13 (8). Based on full-genome molecular comparison, we proposed a new astrovirus genotype species: BoAstV CH13/NeuroS1 (9). The histopathological lesions in these cases were reminiscent of those observed in ESBE. This situation prompted us to investigate brain tissues of those historical cases examined in the original ESBE studies by Fankhauser for BoAstV CH13/NeuroS1. Our results suggest that astroviruses were already involved in the pathogenesis of the disease several decades ago, but have gone undetected.

## Materials and Methods

### Tissue Samples

We selected formalin-fixed paraffin-embedded (FFPE) brain tissues of histologically confirmed cases of ESBE (*n* = 14, Table 1), of bovines without histological evidence of encephalitis (*n* = 8), and of encephalitis cases of other etiology than viral (*n* = 8) from our archives. Tissues included sagittal sections of the brainstem, cerebellum, cerebrum, hippocampus, and additional CNS regions, if available. As the paraffin was friable, tissues were re-embedded in fresh paraffin to be suitable for microtome cutting. All cases showed lesions consistent with the diagnosis of ESBE, which comprise neuronal necrosis, gliosis, and lymphohistiocytic perivascular cuffs. Details on the ESBE cases included in this study are presented in Table 1.

**Table 1:** **Clinical history on cases of European sporadic bovine encephalitis diagnosed between 1958 and 1976**.

Case ID	Year of diagnosis (reference)	Age (years)	Sex	Anamnesis	Duration of disease	Neuropathological diagnosis
3240	1958 (3)	2	F	Severe progressive cerebellar ataxia, dysmetria, hypermetria	6 weeks	Disseminated non-suppurative meningoencephalitis
3466	1959 (2)	1	M	Hypersensitivity, stretched neck and head carriage, inappetence	1 week	Disseminated non-suppurative meningoencephalitis
3628	1959 (2)	2	F	Suspect for malignant catarrhal fever		Non-suppurative meningoencephalitis
3647	1959 (2)	n.k.	M	Excitation, body temperature 40.3°C, heart rate 120 bpm, suspicious for listeriosis	<1 week	Disseminated non-suppurative meningoencephalitis
3812	1960 (2)	6	M	Indifference toward females, stubborn behavior, ataxia, recumbency, tremor, pupillary reflex decreased	~6 months	Disseminated non-suppurative meningoencephalitis
4187	1960 (2)	5	F	Fever, difficulties in rising, later recumbency, opisthotonus, no pain reactions during injections, clinical signs indicative for lesions of the spinal cord	3 days	Non-suppurative meningoencephalitis
4268	1960 (2)	n.k.	F	Stiffness in the back, circling movements, pushing forward, excitation	2 weeks	Non-suppurative encephalitis
11577	1975 (5)	3	F	CNS disease, clonic seizures, salivation	n.k.	Poliencephalitis, cerebellar meningitis
11646	1976 (5)	1.5	F	Rabies suspect	n.k.	Non-suppurative meningoencephalitis
11648	1976 (5)	n.k.	F	Ataxia, collapse, clonic seizures	<1 week	Non-suppurative encephalitis
11697	1976 (5)	2.5	F	CNS disorder, biting the edge of the crib, bangs head against the wall, salivation, intermittent recumbency	3 days	Non-suppurative polio meningoencephalitis
11729	1976 (5)	2.5	F	Raging, biting the edge of the crib und trough, sweating, groaning	<1 week	Non-suppurative meningoencephalitis
11740	1976 (5)	>10	F	Nervous disorder, recumbency, sheep with confirmed rabies on the same farm	1 month	Disseminated non-suppurative meningoencephalitis
11930	1976 (5)	2	F	n.k.	n.k.	Non-suppurative encephalitis

*n.k., not known*.

### Histopathology and *In Situ* Hybridization

Serial sections of 4 μm were cut from FFPE blocks and mounted on SuperFrost Ultra plus glass slides (R. Langenbrinck) for hematoxylin and eosin staining and *in situ* hybridization (ISH). Pathological lesions were evaluated semi-quantitatively as mild, moderate, or severe inflammation.

We applied a non-radioactive digoxigenin-labeled ISH using two distinct probes (A and B) for the detection of BoAstV CH13/NeuroS1 RNA, as described previously (8). ISH-probes A and B were generated by *in vitro* transcription from cloned cDNA with the DIG RNA Labeling Kit (Roche Applied Science). Probe A is complementary to a sequence at the 5′ end of open-reading frame (ORF) 2, whereas probe B is complementary to a sequence in the center of ORF2 of the viral genome. Briefly, tissue sections were deparaffinized, rehydrated, and treated with 0.2M HCl. Proteinase K (Roche) was added to a buffer containing 1M Tris–HCl and 0.1M CaCl_2_, incubated on the slides at 37°C for 15 min and deactivated by 4% (w/v) paraformaldehyde. Next, the slides were treated with a hybridization mix containing 50% (v/v) deionized formamide, 0.05% Ficoll (w/v), 0.05% polyvinylpyrrolidone (w/v), 0.05% BSA (w/v), 4× SSC, and 0.25% yeast RNA (w/v). After 2 h of prehybridization at 50°C, slides were incubated with either of the two ISH probes [1 ng/ml in hybridization mix, supplemented with 0.5% (w/v) yeast RNA and 10% (w/v) dextran sulfate] for 15 h at 50°C. Intense washing and RNAse treatment were performed to enhance binding specificity. Binding of probes was finally detected using anti-digoxigenin-AP-Fab fragments (Roche) and NBT/BCIP substrate (Roche).

### RNA Extraction from FFPE Tissue Blocks

Formalin-fixed paraffin-embedded tissues were cut to 20 μm sections using fresh blades for each sample. The microtome was cleaned with 4% (v/v) hydrogen peroxide after each tissue block. Sections were collected in 1.5 ml tubes and deparaffinized twice with 1 ml xylene at 50°C. After a short centrifugation, the remaining tissue pellet was washed with 1 ml ethanol and incubated with Proteinase K (Roche High pure FFPE RNA Micro Kit) for 3 h at 55°C. RNA was then extracted with Trizol reagent (Thermofisher Scientific) according to the instructions of the manufacturer. RNA quantity and integrity was analyzed by the Fragment Analyzer CE12 (Advanced Analytics).

### Reverse Transcription PCR

RNA was reverse transcribed using the ThermoScript reverse transcription (RT)-PCR kit (Life Technologies) and gene-specific reverse primers MA2 and bAV4, respectively. The resulting cDNA was purified with S300 Microspin columns (GE Healthcare Life Sciences). Two different PCR protocols were applied (i) MA4/MA2 PCR targeting a conserved region of the ORF 1B and (ii) a nested PCR using primers bAV3/bAV4 (first round) and bAV1/bAV2 (second round) targeting a part of the sequence of ORF 2, where ISH probe B is expected to bind. Primer sequences and cycle condition are shown in Table 2. PCR was performed with the GoTaq Green Mastermix Green (Promega) according to the instructions of the manufacturer.

**Table 2:** **Details on primers and settings for BoAstV-CH13/NeuroS1 RT-PCR**.

PCR		Primer	Sequence (5′–3′)	Conditions
ORF 1B RT-PCR	MA2/MA4	MA2	GGC TTT ACC CAC ATI CCA AA	95°C, 2 min
		MA4	TGG ACC CGC TAT GAT GGC ACI AT	5× (95°C, 30 s; 55°C, 30 s; 72°C, 1 min)
				5× (95°C, 30 s; 53°C, 30 s; 72°C, 1 min)
				30× (95°C, 30 s; 51°C, 30 s; 72°C, 1 min)
				72°C, 7 min
ORF2 nested RT-PCR	bAV3/bAV4	bAV 3	ACC GCC TTT CCG ATG ATG TGC	95°C, 2 min
		bAV 4	TTC ATC AAC AAC CTG CCA TAT	39× (95°C, 15 s; 52°C, 15 s; 72°C, 30 s)
				72°C, 10 min
	bAV1/bAV2	bAV 1	GAT TCT GAG GGC CAA ATA ACC	95°C, 2 min
		bAV 2	GCC AAA TGG TCT CCC CAA CAG	39× (95°C, 15 s; 52°C, 15 s; 72°C, 30 s)
				72°C, 10 min

### Cloning and Sequencing

Deoxyadenosine was added to the 3′ ends of the amplicons by incubation of the PCR reaction with 0.5 μl Taq DNA polymerase (New England Biolabs). The PCR reaction was separated on 1% agarose gels, with bands extracted using the Wizard SV Gel Extraction and PCR clean-up Kit (Promega). Amplicons were then cloned into pCR4 Topo vector (Life Technologies), and the resulting plasmid was transformed into One Shot Top10 cells (Life Technologies). Colonies were picked and cultured in 1 ml LB plus ampicillin at 37°C in a shaker incubator at 220 rpm. Plasmids were extracted with the PureYield plasmid mini preparation kit (Promega). Insert sequencing was carried out with primers M13 forward and M13 reverse according to the BigDye terminator sequencing protocol (Thermofisher Scientific). Sequences were analyzed with Molecular Evolutionary Genetics Analysis software (MEGA, version 6.0).

## Results

### Detection of BoAstV CH13/NeuroS1

**Figure 1:**
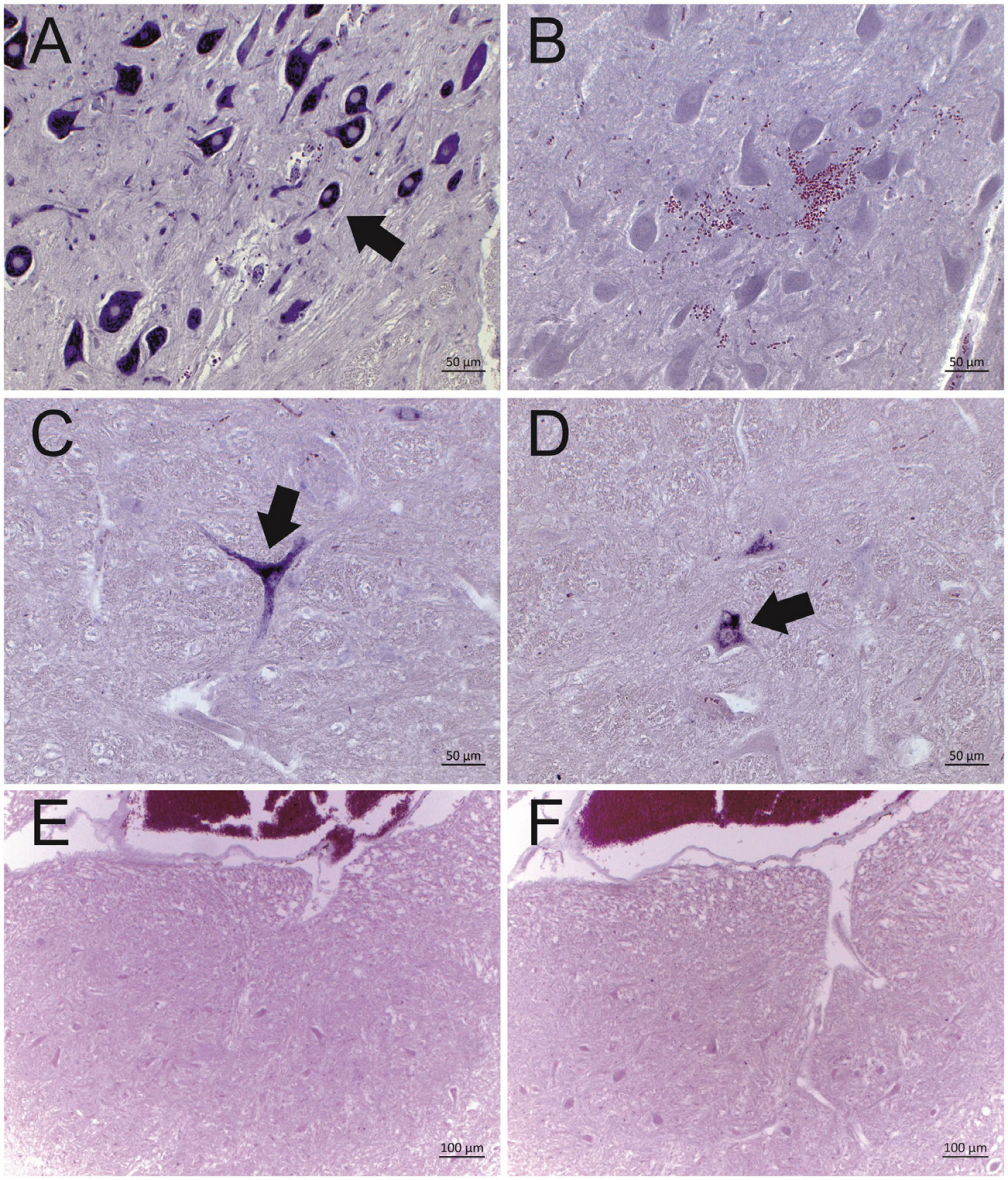
**Representative results of the *in situ* hybridization (ISH) for BoAstV-CH13/NeuroS1 RNA in cattle brain tissues**. Two DIG labeled RNA probes [probe A: micrographs **(A,C,E)**; probe B, micrographs **(B,D,F)**] were applied. Arrows indicate individual ISH-positive neurons. **(A,B)** Historical case of European sporadic bovine encephalitis (case 3628), which was diagnosed in 1959. Note that only probe A was reactive, but not probe B. **(C,D)** BoAstV-CH13/NeuroS1 positive control case, dating from 1995. Both probes show a clear labeling of neurons. **(E,F)** Negative control tissues with the absence of labeling by both probes.

Twelve ESBE cases revealed a positive deep purple staining in at least one of the tested brain regions with ISH probe A. However, none of the cases showed positive labeling with probe B. Negative control tissue sections analyzed in parallel in each run (Figure 1), as well as the brain tissues of bovines without histological lesions (*n* = 8) and with encephalitis of other etiologies than viral (*n* = 8), remained unlabeled with both probes. Collectively, these data confirm the presence of astrovirus RNA in the brains of 85% (12/14) of the historical cases of ESBE. In these animals, the astrovirus RNA could be detected in different regions of the central nervous system, but the distribution pattern was variable between cases (Table 3). The brainstem was astrovirus positive in the majority of the cases, but mostly single neurons were labeled, whereas in the hippocampus, the infection tended to be more severe, affecting almost all cells of the pyramidal and molecular layer. However, the limited number of cases and the limited availability of brain regions for some cases of non-suppurative encephalitis do not allow further conclusions on the virus distribution.

**Table 3:** **Details on historical cases of European sporadic bovine encephalitis diagnosed between 1958 and 1976 in Switzerland**.

Case ID	BoAstV CH13/NeuroS1	Brain region (ISH score/lesion score)				
		Brainstem	Cerebellum	Cerebrum	Hippocampus	Other
3240	positive	3/3	0/0.5	–	–	–
3466	positive	–	2/2	1/2	0/1.5	1/2
3628	positive	3/3^a^	–	3/3	–	–
3647	negative	0/3	0/2	0/2	0/3	–
3812	positive	3/2	0/1	–	–	0/2.5
4187	positive	1/3	0/0	–	–	1/2.5
4268	negative	0/2.5	0/2	–	–	0/2.5
11577	positive	3/2.5	3/2	1/3	3/1.5	–
11646	positive	–	0/3	0/2	3/1.5	0/2.5
11648	positive	–	1/0^a^	2/0	3/1.0	3/3^b^
11697	positive	–	1/1.5	0/2	3/2.5^a^	2/3^a^
11729	positive	0/3	0/2	3/2	–	–
11740	positive	2/2	0/0.5^a^	1/1	–	2/2
11930	positive	2/2.5^b^	1/2^a^	–	–	–

*Different brain regions were investigated for the severity of histopathological lesions and the presence of bovine astrovirus RNA*.

*BoAstV CH13 *in situ* hybridization (ISH) score: 0 = no positive cells; 1 = 1–10 positive cells; 2 = 10–50 positive cells, 3 = >50 positive cells; lesion score: 0 = no lesions, 0.5–1 = mild lesions, 1.5–2 = moderate lesions, 2.5–3 = severe lesions; topographical correlation of lesions and BoAstV CH13: ^a^partial correlation, ^b^good correlation; –, not available*.

### Correlation of BoAstV CH13/NeuroS1 RNA and Histopathological Brain Lesions

**Figure 2:**
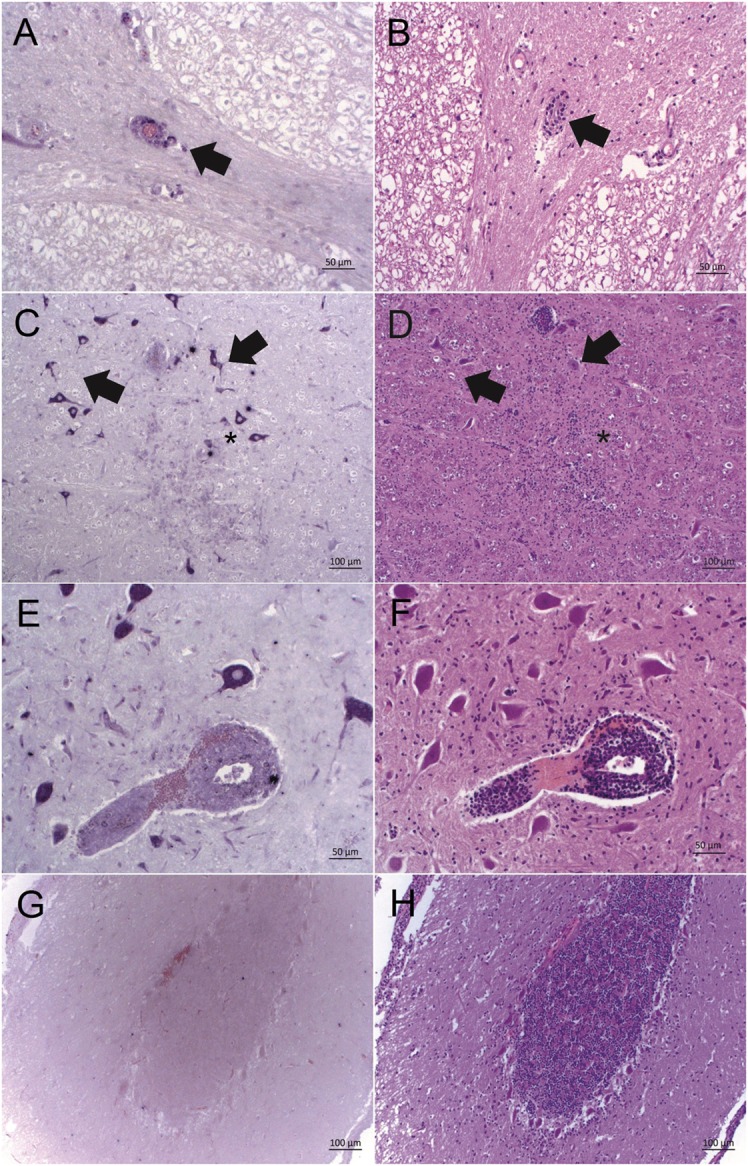
**Bovine astrovirus RNA and its correlation with histopathological lesions in brain tissues of historical cases of European sporadic bovine encephalitis**. Astrovirus RNA was detected by *in situ* hybridization with probe A [ISH, purple labeling; **(A,C,E,G)**] and histopathological lesions were assessed after hematoxylin and eosin staining [HE; **(B,D,F,H)**]. **(A,B)** Case 11930, brainstem: good correlation of ISH labeling and histopathological lesions in perivascular cuffs (arrow). **(C,D)** Case 3628, brainstem: strong ISH labeling in neurons in vicinity of lesions (arrows), and weak ISH labeling of glia cells in a glial node (asterisk). **(E,F)** Case 3628, brainstem: strongly ISH labeled neurons and weakly labeled mononuclear cells in a perivascular cuff. **(G,H)** Case 11646, cerebellar cortex: lack of correlation between lesions and ISH labeling. While there is a moderate gliosis, cells remain ISH negative. Magnifications of microphotographs are indicated by scale bars.

To further investigate whether astrovirus positive cells were topographically related to histopathological lesions, ISH labeling was assessed semi-quantitatively based on the number of ISH labeled cells. The ISH labeling was in most cases confined to neurons, as judged by the morphological appearance of the cells. Interestingly, in case 11930, we detected a positive staining in mononuclear cells of a perivascular cuff in addition to the neurons (Figures 2A,B), and in case 3628, cells in a glial node were reactive with the ISH probe as well (Figures 2C,D). In all other cases, astrovirus RNA was found predominantly in neurons, but without a clear association to neuronal necrosis, gliosis, or perivascular infiltrations. In three animals, labeled neurons were found in proximity to histological lesions (Figures 2E,F). However, in some animals, astrovirus RNA was detected in neurons without any association to lesions, and vice versa, in many areas with lesions, the cells were devoid from astrovirus RNA (Figures 2G,H).

### Sequence Analysis

**Figure 3:**
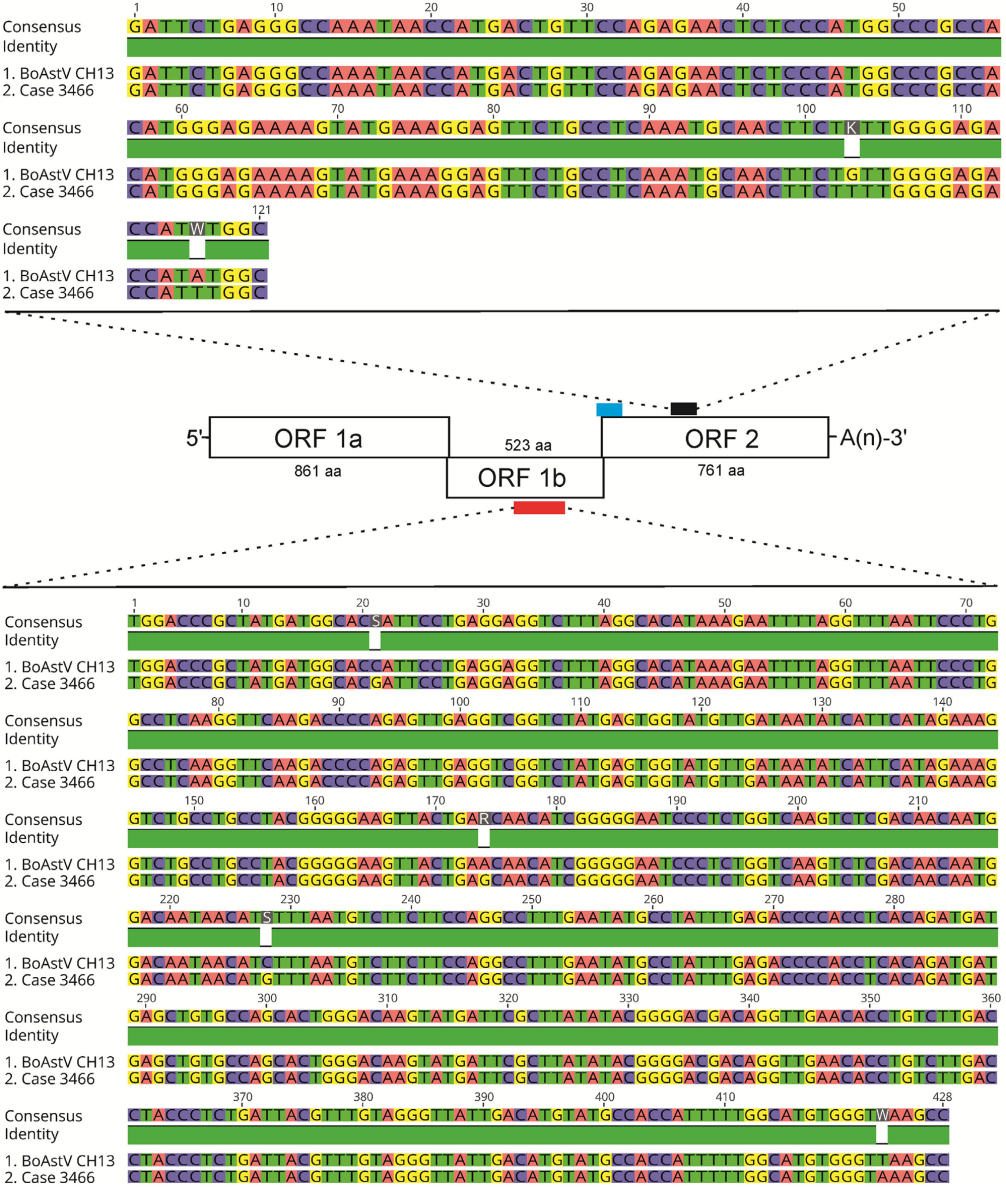
**Results of RT-PCR and sequencing of astrovirus RNA extracted from case 3466**. A scheme of the BoAstV CH13 genome is presented. The viral genome is organized in three open-reading frames (ORF). The red bar indicates the target sequence for RT-PCR using primers MA2/MA4 within ORF 1b, which encodes for the RNA-dependent RNA polymerase. The blue and black bars show target sequences of *in situ* hybridization probes A and B, respectively, within ORF2 that encodes for structural capsid proteins. The nested RT-PCR protocol with primers bAV3/bAV4 (first round) and bAV1/bAV2 (second round) was designed to yield amplicons in the target region of ISH probe B (black bar). Sequence comparisons of the MA2/MA4 and of the bAV1/bAV2 amplicons of case 3466 with the BoAstV CH13 reference sequence (GenBank accession number: NC_024498) are shown. Alignments were generated with the Geneious R9 software, version 9.0.4 (Biomatters).

The discrepancy in the reactivity of ISH probe A and B in ESBE cases was unexpected, since more recent BoAstV CH13/NeuroS1 positive tissues (8), as well as the positive control tissues used in the present study, were invariably reactive with both probes (Figures 1C,D). This raised the possibility that the historical cases had been infected with an astrovirus different to BoAstV CH13/NeuroS1. To address this point, we extracted total RNA from FFPE tissue and performed two different RT-PCR protocols. Despite several attempts, RNA yields for most FFPE tissues were below the detection limit (as assessed by Fragment Analyzer CE12), and RT-PCRs did not yield amplicons. Yet in one animal (case 3466), we were able to obtain amplicons by both RT-PCR protocols. Amplicon sequences were >98% identical to those of BoAstV CH13/NeuroS1, confirming that the target region for ISH probe B shows no significant difference in sequence analysis and that this animal was very likely to have been infected with BoAstV CH13/NeuroS1 (Figure 3).

## Discussion

We found astrovirus RNA in the brains of a high proportion of historical ESBE cases, but not in control animals, which underpins the association with the disease. The astrovirus is present in different brain regions, affecting not only neurons but also inflammatory cells in perivascular cuffs and glial nodes, without revealing a clear neuroanatomical distribution pattern.

Generally, the microtopographical localization of the viral RNA did not match that of the histopathological lesions in the affected brains. This could be due to specific disease pathomechanisms: the immune response can lead to rapid clearance of the virus in inflammatory lesions, as known for tick-borne encephalitis (10). However, it could also indicate that the astrovirus is neuroinvasive, but with limited neurovirulence, and that other yet unidentified viruses could play a role as cofactors. Indeed, there has been evidence for a paramyxovirus in brain tissues of cases of ESBE (6, 11), and very recently, we have reported the second neurotropic astrovirus (BoAstV CH15) in two cows with non-suppurative encephalitis in Switzerland (12). More comprehensive studies aiming at identifying disease-associated pathogens beyond BoAstV CH13/NeuroS1 in cases of bovine encephalitis have identified additional candidate viruses, such as parainfluenzavirus 5, bovine polyomavirus 2, and bovine herpesvirus 6, which might be involved in the etiology of non-suppurative encephalitis in cattle as well (13). At the moment, we have not yet established the tools to detect these viruses in historical FFPE tissues; however, it will be interesting to investigate such cases for potential coinfections with BoAstV-CH/NeuroS1.

Long-term storage of tissues, re-embedding in fresh paraffin, and loss of RNA integrity may account for negative RT-PCR results and for the failure in detecting viral RNA by ISH probe B. Still, the virus likely corresponds to BoAstV CH13/NeuroS1, because ISH probe A was specifically designed for this virus, and we recovered BoAstV CH13/NeuroS1 sequences in one of these animals by RT-PCR. However, we cannot exclude the possibility that ISH-positive cases with unconfirmed virus sequences might be infected with a different neurotropic astrovirus with sequence similarities to BoAstV-CH13/NeuroS1 in the binding region of ISH probe A. This may, in particular, be applicable in the two cases in which the IHC labeling indicated virus infection in cells other than neurons.

Viral encephalitis of unknown origin has been a frequent diagnosis in cattle with neurological disease ever since neuropathologists such as Frauchiger and Fankhauser started to investigate such cases. Now, we are increasingly gaining knowledge on possible etiologies, among which neurotropic astroviruses are to be found. Our results provide evidence that astroviruses were involved in the pathogenesis of bovine poliencephalitis decades ago and serve as a prerequisite for understanding the pathogenesis of so far unresolved viral neuroinfectious diseases.

## Author Contributions

TS, AO, and MV designed the study. IB and SS-H performed the experiments. SS-H, AO, and MV assessed the pathology. SS-H, MV, AO, and TS wrote the manuscript.

## Conflict of Interest Statement

The authors declare that the research was conducted in the absence of any commercial or financial relationships that could be construed as a potential conflict of interest.
